# The Absence of Mrp4 Has No Effect on the Recruitment of Neutrophils and Eosinophils into the Lung after LPS, Cigarette Smoke or Allergen Challenge

**DOI:** 10.1371/journal.pone.0061193

**Published:** 2013-04-22

**Authors:** Jürgen Schymeinsky, Hannah Mayer, Christopher Tomsic, Cornelia Tilp, John D. Schuetz, Yunhai Cui, Lutz Wollin, Florian Gantner, Klaus J. Erb

**Affiliations:** 1 Respiratory Diseases Research, Boehringer Ingelheim Pharma GmbH & Co. KG, Biberach a.d. Riss, Germany; 2 Department of Pharmaceutical Sciences, St. Jude Children's Research Hospital, Memphis, Tennessee, United States of America; French National Centre for Scientific Research, France

## Abstract

The multidrug resistance protein 4 (Mrp4) is an ATP-binding cassette transporter that is capable of exporting the second messenger cAMP from cells, a process that might regulate cAMP-mediated anti-inflammatory processes. However, using LPS- or cigarette smoke (CS)-inflammation models, we found that neutrophil numbers in the bronchoalveolar lavage fluid (BALF) were similar in *Mrp4^−/−^* and *Mrp4^+/+^* mice treated with LPS or CS. Similarly, neutrophil numbers were not reduced in the BALF of LPS-challenged wt mice after treatment with 10 or 30 mg/kg of the Mrp1/4 inhibitor MK571. The absence of Mrp4 also had no impact on the influx of eosinophils or IL-4 and IL-5 levels in the BALF after OVA airway challenge in mice sensitized with OVA/alum. LPS-induced cytokine release in whole blood *ex vivo* was also not affected by the absence of Mrp4. These data clearly suggest that Mrp4 deficiency alone is not sufficient to reduce inflammatory processes *in vivo*. We hypothesized that in combination with PDE4 inhibitors, used at suboptimal concentrations, the anti-inflammatory effect would be more pronounced. However, LPS-induced neutrophil recruitment into the lung was no different between *Mrp4^−/−^* and *Mrp4^+/+^* mice treated with 3 mg/kg Roflumilast. Finally, the single and combined administration of 10 and 30 mg/kg MK571 and the specific breast cancer resistance protein (BCRP) inhibitor KO143 showed no reduction of LPS-induced TNFα release into the BALF compared to vehicle treated control animals. Similarly, LPS-induced TNFα release in murine whole blood of Mrp4^+/+^ or Mrp4^−/−^ mice was not reduced by KO143 (1, 10 µM). Thus, BCRP seems not to be able to compensate for the absence or inhibition of Mrp4 in the used models. Taken together, our data suggest that Mrp4 is not essential for the recruitment of neutrophils into the lung after LPS or CS exposure or of eosinophils after allergen exposure.

## Introduction

The multidrug resistance protein 4 (Mrp4, ABCC4) is a member of the large family of ATP-binding cassette (ABC) transporters that are required for the active transport of substrates across the cell membrane [1]. The export of endogenous and xenobiotic substrates, ranging from ions to macromolecules, through Mrp4 works even against a chemical gradient by using the energy of ATP hydrolysis [2]. Mrp4 is expressed in various blood cells, neurons, and in epithelial and endothelial cells, where it can be localized to either the basolateral or the apical membrane [2]. Constitutive absence of Mrp4 reveals *Mrp4^−/−^* mice are fertile and healthy which shows Mrp4 is not essential for life [3]. Mrp4 transports a broad range of physiologic substrates that include molecules with key roles in cellular signaling processes like the cyclic nucleotides cAMP and cGMP (cyclic adenosine and guanosine monophosphate), ADP (adenosine diphosphate), eicosanoids, urate and steroid hormones [2], [4], [5]. Mrp4 is also able to confer resistance to certain therapeutic drugs, e.g. the anticancer agent Topotecan [2], [3], [6]. Thus, the inhibition of Mrp4 may improve the therapeutic efficacy of some drugs.

Mrp4 may be involved in the regulation of the intracellular amount of cAMP concentrations. This second messenger has a key role in different cellular functions, including the regulation of the endothelial barrier, the contraction of smooth muscle cells and the activation of inflammatory cells [2], [7]–[9]. The ability of Mrp4 to regulate intracellular cAMP was demonstrated in HT-29 gut epithelial cells, where treatment with either a Mrp1/4 inhibitor MK571 or a Mrp4-specific siRNA significantly increased cAMP levels upon stimulation with adenosine [10]. Similarly, higher cAMP intracellular to extracellular ratios were detectable in human coronary artery smooth muscle cells upon the downregulation of Mrp4 by siRNA technique indicating that Mrp4 could also have a regulatory effect on basal intracellular cAMP levels [11].

The second messenger cAMP is generated by the activity of adenylyl cyclases that are mainly activated by G protein coupled receptors, like the β_2_ adrenergic receptors [12]. In mammalian cells there are at least three known types of cAMP effector proteins: protein kinase A (PKA), exchange proteins activated by cAMP (EPACs), and cyclic nucleotide gated ion channels (CNGs) [13]. Current strategies to enhance the intracellular cAMP level for the treatment of inflammatory lung diseases include the inhibition of cyclic nucleotide phosphodiesterase 4 (PDE4) [14]–[16], which catalyzes the degradation of cAMP, and the activation of the β_2_ adrenergic receptor [17]. However, PDE4 is expressed also in the brain and its inhibition often induces centrally-mediated emesis and increased cAMP levels in neurons are generally associated with increased nociception [12], [18]–[20]. Based on the function of Mrp4 and the relevance of cAMP for the inflammatory processes, we postulated that inhibition of Mrp4 may be a new approach to dampen inflammation.

The present study was undertaken to test whether the absence of Mrp4 has a beneficial effect in different murine models of chronic obstructive pulmonary disease (COPD) and asthma. In addition, we tested if the absence of Mrp4 would have an additive effect on PDE4 inhibitors in the reduction of the inflammatory responses. We analyzed Mrp4-deficient mice in comparison to wt controls in models of lipopolysaccharide- (LPS-), ovalbumin- (OVA-) and cigarette smoke-induced lung inflammation. Furthermore, we compared the potency of the PDE inhibitor Roflumilast between *Mrp4^−/−^* and *Mrp4^+/+^* mice in the model of LPS-induced neutrophil recruitment into the lung. Since the ABC transporter BCRP (breast cancer resistance protein, ABCG2) shares some substrates with Mrp4, including cAMP, we tested the possibility whether Mrp4 deficiency could be compensated by BCRP by using a specific inhibitor KO143 [21], [22] in a model of LPS-induced TNFα release in murine whole blood. Finally, we tested in an *in vivo* pharmacological approach the effects of the orally available inhibitors MK571 (Mrp1/4 inhibitor) [23], [24] and KO143 on neutrophil recruitment and cytokine release in wt mice treated with LPS.

## Methods

### Mice

The generation of Mrp4 deficient mice used in this study has been described elsewhere [3]. Animals were maintained under conventional conditions in an isolation facility. At the onset of the experiments, animals were between 6 and 8 weeks of age. All experiments were performed according to the guidelines of the local and government authorities for the care and use of experimental animals (Regierungspräsidium Baden-Württemberg, Tübingen, Germany; approval numbers: 05-005, 08-004, 08-005 and 12-014).

### Measurement of cytokine and cAMP levels in plasma or BALF of mice

Blood of *Mrp4^+/+^* and *Mrp4^−/−^* mice was collected retro-bulbar using heparin-treated glass capillaries (Microvette 500 LH, Sarstedt) during short-term anesthesia by inhalation of isoflurane (3–4%). To induce cytokine release, 600 ng/ml Lipopolysaccharide (LPS; *E. coli* 055B5, Sigma) or PBS for control was added to the blood samples and incubated for 4 h at 37°C, 5% CO_2_. Plasma was collected after centrifugation and frozen at −80°C. Multiplex analysis was performed by using the MILLIPLEX Mouse Cytokine/Chemokine Kit Premixed 22 Plex kit (Millipore) according to the manufacturer's protocol. The samples were measured using a Bio-Plex system and analyzed by Bio-Plex software (Bio-Rad). For quantification of cAMP plasma levels the MSD (Meso Scale Discovery) Cyclic AMP Assay Kit was used according to the manufacturer's protocol and measured with the SECTOR® Imager 6000 (Meso Scale Discovery). Murine TNFα, IL-4 or IL-5 concentrations in the BALF or plasma, respectively, were measured by using BD Pharmingen ELISA kits (BD OptEIA™ Mouse TNF/IL-4/IL-5 ELISA Kit) or MSD Mouse TH1/TH2 9-Plex Ultra-Sensitive Kits according to the manufacturers' protocols.

### Bronchoalveolar lavage

At indicated times, mice were sacrificed by intraperitoneal pentobarbital injection (400 mg/kg, Narcoren, Merial GmbH, Halbergmoos, Germany), blood samples collected, the trachea cannulated and a bronchoalveolar lavage (BAL) performed by flushing the lungs and airways 2 times with 1 ml Hanks salt solution (Biochrom AG). Total cell count of BAL cells and differential cell count were determined by means of a Sysmex XT1800 iVet cell analyzer (Sysmex Europe GmbH, Norderstedt, Germany) utilizing customized settings that were validated with cytospin preparations (data not shown).

### Reference compound administration

Dexamethasone, Rolipram, MK571 and Ko143 (Sigma–Aldrich) or Roflumilast (resynthesized at Boehringer Ingelheim Pharma GmbH & Co. KG, Biberach, Germany) at indicated doses were suspended in hydroxyethylcellulose 0.5–1.0% (Merck) and administered orally by gavage (10 ml/kg body weight).

### Intratracheal treatment of mice with lipopolysaccharide

The mice received short-term anesthesia by inhalation of isoflurane (3–4%). Mice were fixed in supine position on an inclined operating table. A 22G plastic catheter (Vasofix, Braun) was inserted into the trachea guided by a fiber optic light. LPS (*E. coli* 0111:B4, Sigma), 5 µg in 50 µl of aqueous NaCl 0.9% was administered intratracheally by means of a 1 ml syringe.

### Induction of OVA-specific Th2 responses

For the induction of OVA-specific Th2 responses a recently described protocol was used [25]. Briefly, mice were treated three times i.p. with a mixture of 20 µg OVA (Serva) in 200 µl alum adjuvant solution (Thermo Scientific) on the days 0, 14, and 21. Subsequently on day 26 and 27, mice were challenged with aerosolized OVA (1% in PBS) for 20 min. Bronchoalveolar lavage was performed on day 28.

### Pulmonary inflammation upon exposure to cigarette smoke

The protocol for the induction of acute inflammation upon cigarette smoke (CS) exposure has been used and described previously [26]. Briefly, the mice were exposed to CS for 4 days in a whole body exposure box that was heated (38°C) to maintain the physiological body temperature of the animals. On day one and two, mice were exposed to the mainstream smoke of 6 cigarettes (Roth-Händle without filters, tar 10 mg, nicotine 1.0 mg, carbonmonoxide 6 mg, Badische Tabakmanufaktur Roth-Händle®), of 8 cigarettes on day 3, and of 10 cigarettes on day 4. Exposure to the smoke of each cigarette lasts for 15 min (cigarette was completely burned in the first two minutes by an airflow of 3.3 l/min) followed by 8 min exposure with fresh room air (15 l/min). Every second cigarette an additional break of 24 min with exposure to fresh room air (15 l/min) was conducted. A semi-automatic cigarette lighter and smoke generator with an electronic timer was used to control the CS exposure (Boehringer Ingelheim Pharma GmbH & Co. KG, Biberach, Germany). CS particle concentration was monitored by a real time ambient particle monitor (MicroDust Pro, Casella). Control animals were exposed to room air.

### Statistical analysis

The inter- and intra-experimental deviations of the *in vivo* and *ex vivo* experiments were in the expected and usual range of the validated models. Results were evaluated using the unpaired Student's t test or one-way ANOVA with subsequent parametric Dunnett's multiple comparison test for cellular data and nonparametric Kruskal–Wallis test for mediator release data. Statistical significance was accepted at p<0.05. The GraphPad Prism software (version 5.04, GraphPad Software, Inc) was used for statistical analyses.

## Results

### LPS-induced neutrophil accumulation in the lung was not affected in *Mrp4^−/−^* mice or in wt mice treated with MK571

**Figure 1:**
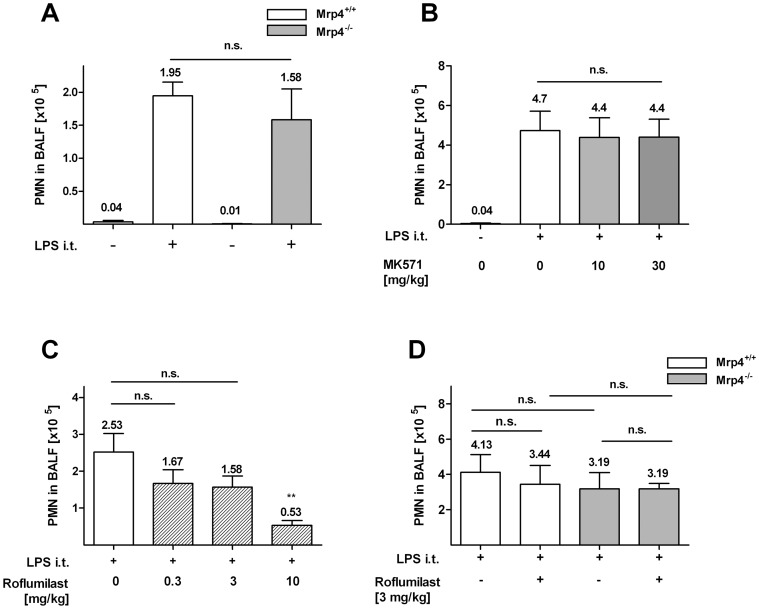
LPS-induced neutrophil influx into the lung was similar in *Mrp4* ^−/−^ and *Mrp4* ^+/+^ mice and in wt mice treated with MK571. A–D) Neutrophil concentration in the BALF was measured 4 h after intratracheal application of LPS (+; 50 µl of a 100 µg/ml solution) or PBS for control (−). A) The application of LPS induced neutrophil infiltration into the lungs of *Mrp4^+/+^* (open white bar) and *Mrp4^−/−^* (open grey bar) mice compared to PBS treated control animals. B) Wt mice were treated p.o with doses of 10 or 30 mg/kg MK571 or C) with p.o. doses of 0.3, 3 or 10 mg/kg Roflumilast or B–C) with PBS for control (0 mg/kg). Neutrophil numbers were measured after 4 h LPS stimulation. B) The inhibition of Mrp4 and Mrp1 with MK571 had no effect on neutrophil recruitment. C) At the highest dose of 10 mg/kg Roflumilast neutrophil influx was significantly reduced compared to untreated controls. D) 20 min before the LPS-challenge, *Mrp4^+/+^* (white bar) and *Mrp4^−/−^* mice (grey bar) were treated with a suboptimal concentration of 3 mg/kg Roflumilast (+) or PBS for control (−). After 4 h no significant difference in PMN recruitment was detectable between the BALF of *Mrp4^+/+^* and *Mrp4^−/−^* mice with and without 3 mg/kg Roflumilast. n = 5–8. n.s. = not significant. Data are mean ± SEM.

Excessive recruitment of neutrophils into the lung is a hallmark of COPD [27]. The instillation of LPS, a component from the outer cell membrane of gram-negative bacteria, into the lung induces massive neutrophil recruitment which serves as an animal model for COPD [28], [29]. As expected, intra-tracheal LPS application elicited a prominent increase of neutrophil numbers in the BALF of *Mrp4^+/+^* mice (1.95×10^5^ neutrophils/ml) and *Mrp4^−/−^* mice (1.58×10^5^ neutrophils/ml; Figure 1A). However, no difference in neutrophil numbers was detectable between *Mrp4^+/+^* and *Mrp4^−/−^* mice indicating that absence of Mrp4 has no effect on LPS-induced neutrophil recruitment into the lung. Using a pharmacological *in vivo* approach, we orally administered the Mrp1/4 inhibitor MK571 [23] to C57Bl/6 wt mice before the i.t. LPS challenge. *In vivo* efficacy of MK571 has been shown previously at doses of 5 and 25 mg/kg in a murine model of hypoxia-induced pulmonary hypertension [30]. As described above, PMN numbers in the BALF increased significantly upon stimulation with LPS (4.7×10^5^ neutrophils/ml; Figure 1B). In accordance with the results from Mrp4^−/−^ mice, the treatment MK571 did not reduce neutrophil numbers in the BALF confirming that Mrp4 is not involved in the regulation of neutrophil recruitment after LPS challenge.

In the following *in vivo* experiments we used the recently developed PDE4 inhibitor Roflumilast which does not inhibit Mrp4 at 10 µM (data not shown). Roflumilast has been approved for marketing in Europe and the USA as a therapeutic option for treating COPD [31]. It has been demonstrated that Roflumilast inhibits LPS-driven lung inflammation in Wistar rats [32]. Accordingly, the oral application of Roflumilast led to a dose dependent reduction of neutrophil infiltration into the lung of C57BL/6 wt mice (Figure 1C). Compared to untreated controls (2.53×10^5^ neutrophils/ml) a tendency toward reduction of neutrophil influx was detectable at doses of 0.3 and 3 mg/kg Roflumilast (1.67×10^5^ and 1.58×10^5^ neutrophils/ml, respectively) with a significant decrease in neutrophil numbers (0.53×10^5^ neutrophils/ml) at a dose of 10 mg/kg.

To test if Mrp4 absence would increase the efficacy of Roflumilast, we treated *Mrp4^+/+^* and *Mrp4^−/−^* mice with a suboptimal dose of 3 mg/kg Roflumilast (leading to about 50% reduction in neutrophil numbers) 20 min before application of LPS (Figure 1D). However, we could not detect a difference in neutrophil numbers in the BALF between *Mrp4^+/+^* and *Mrp4^−/−^* mice after treatment with Roflumilast (3.44×10^5^ and 3.19×10^5^ neutrophils/ml, respectively) indicating that Mrp4 absence does not have an effect on its own, nor does it have an additive effect with PDE4 inhibitors on LPS-induced neutrophil recruitment into the lung.

### OVA-specific Th2 responses were similar in *Mrp4^−/−^* and *Mrp4^+/+^* control mice

**Figure 2:**
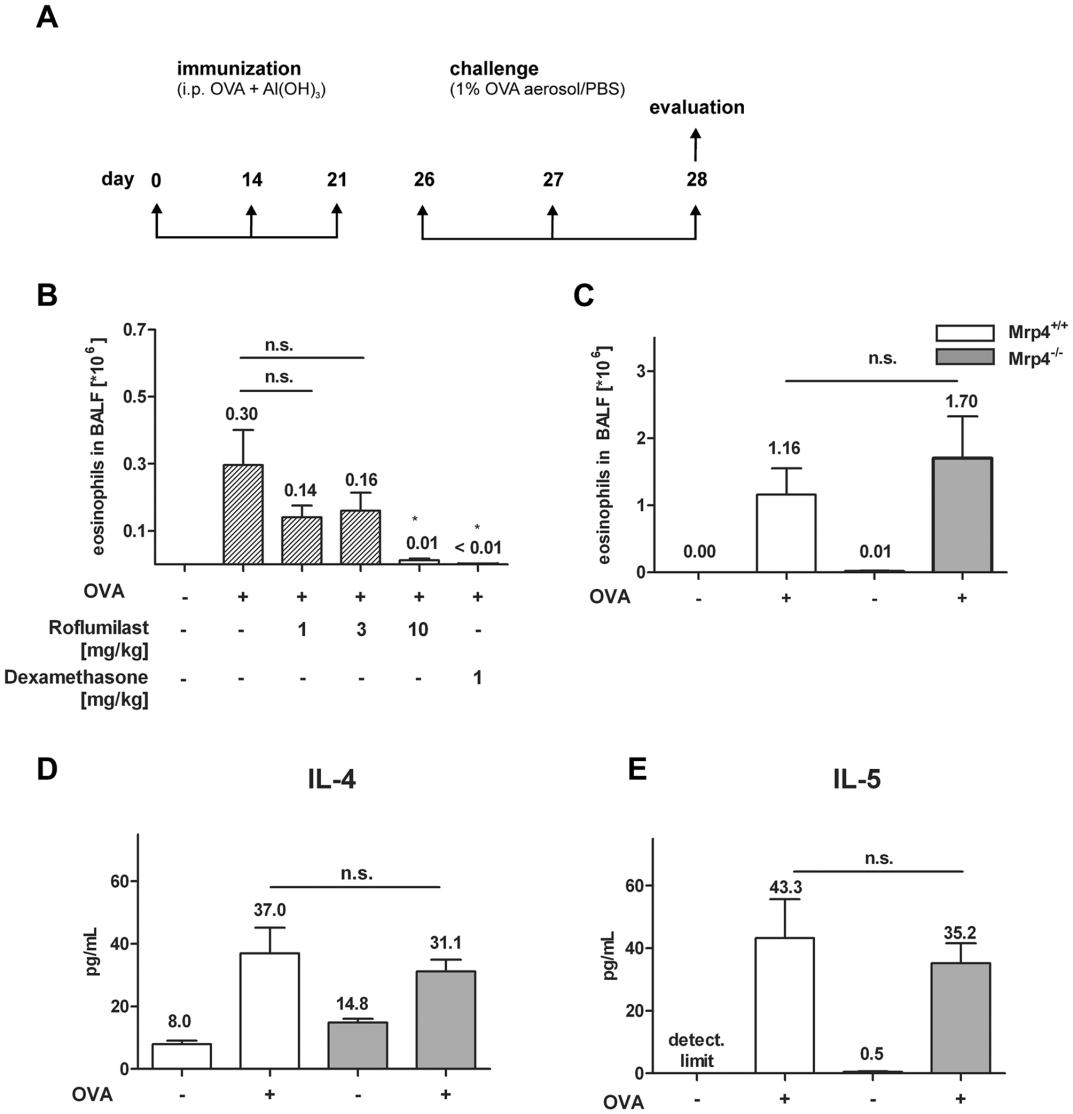
Eosinophil numbers were similar in BALF of OVA-sensitized *Mrp4^−/−^* and *Mrp4^+/+^* mice after OVA-challenge. A) Schematic of the used protocol to induce Th2 mediated lung inflammation. Mice were sensitized by intraperitoneal injections of OVA and the adjuvant Al(OH)_3_ at days 0, 14 and 21. At day 26 and 27 mice were challenged with aerosolized OVA (+) or PBS for control (−) and BALF was collected 1 day after last challenge. B) Eosinophil numbers in the BALF of wt mice after the oral application of the indicated doses of Roflumilast or Dexamethasone (1, 3 or 10 mg/kg) in comparison to PBS treated mice (−) for control. C) Eosinophil numbers or D) IL-4 and E) IL-5 concentrations in the BALF were measured in OVA-sensitized *Mrp4^+/+^* (white bars) or *Mrp4^−/−^* mice (grey bars) after OVA-challenge (+) or PBS control (−). n = 7–8. n.s. = not significant. Data are mean ± SEM.

Next, we investigated whether PDE4 inhibition by Roflumilast and/or the absence of Mrp4 have a suppressive effect on OVA-induced T helper cell type 2 (Th2)-responses in OVA sensitized and lung challenged mice. This animal model mimics some of the pathological symptoms of allergic asthma. OVA sensitized mice were challenged with an aerosol of OVA on two consecutive days and BALF was collected one day after the last allergen challenge (Figure 2A). In a first set of experiments we tested the efficacy of the PDE4 inhibitor Roflumilast in our model of OVA-induced eosinophil recruitment in C57BL/6 wt mice. In accordance with the literature [33], the oral treatment of the mice with 1, 3 and 10 mg/kg Roflumilast once daily during the OVA-challenge reduced the eosinophil numbers in the BALF in a dose-dependent manner (3v10^5^ eosinophils/ml in the untreated control animals, 1.4v10^5^, 1.6×10^5^ and 0.1×10^5^ eosinophils/ml, respectively, in the Roflumilast-treated mice, Figure 2B). The effect of 10 mg/kg Roflumilast was similar to 1 mg/kg of the synthetic glucocorticoid Dexamethasone, consistent with PDE4 inhibition and the associated regulation of the intracellular cAMP concentration required for the inhibition of the eosinophil recruitment in Th2-mediated lung inflammation. However, the eosinophil concentration in the BALF of *Mrp4^−/−^* mice after OVA-challenge suggested that the absence of Mrp4 does not have a suppressive effect on Th2-induced inflammatory responses (Figure 2C). Likewise, the absence of Mrp4 did not affect the release of the cytokines IL-4 and IL-5 (Figure 2D and 2E), which both play an essential role during allergic asthma [27]. Upon challenge with OVA the IL-4 concentration in the BALF increased in *Mrp4* ^+/+^ mice from 8.0 pg/ml to 37.0 pg/ml which was similar in *Mrp4^−/−^* mice, where the IL-4 levels increased from 14.8 pg/ml to 31.1 pg/ml. Similarly, the IL-5 levels upon OVA-stimulation were not significantly different between *Mrp4^+/+^* mice (43.3 pg/ml) and *Mrp4^−/−^* mice (35.2 pg/ml). Taken together, we could not detect any suppressive effect of the absence of Mrp4 on the development of Th2-dependent inflammatory responses.

### Neutrophil recruitment after smoke exposure for 4 days was not decreased in *Mrp4* ^−/−^ compared to *Mrp4* ^+/+^ mice

**Figure 3:**
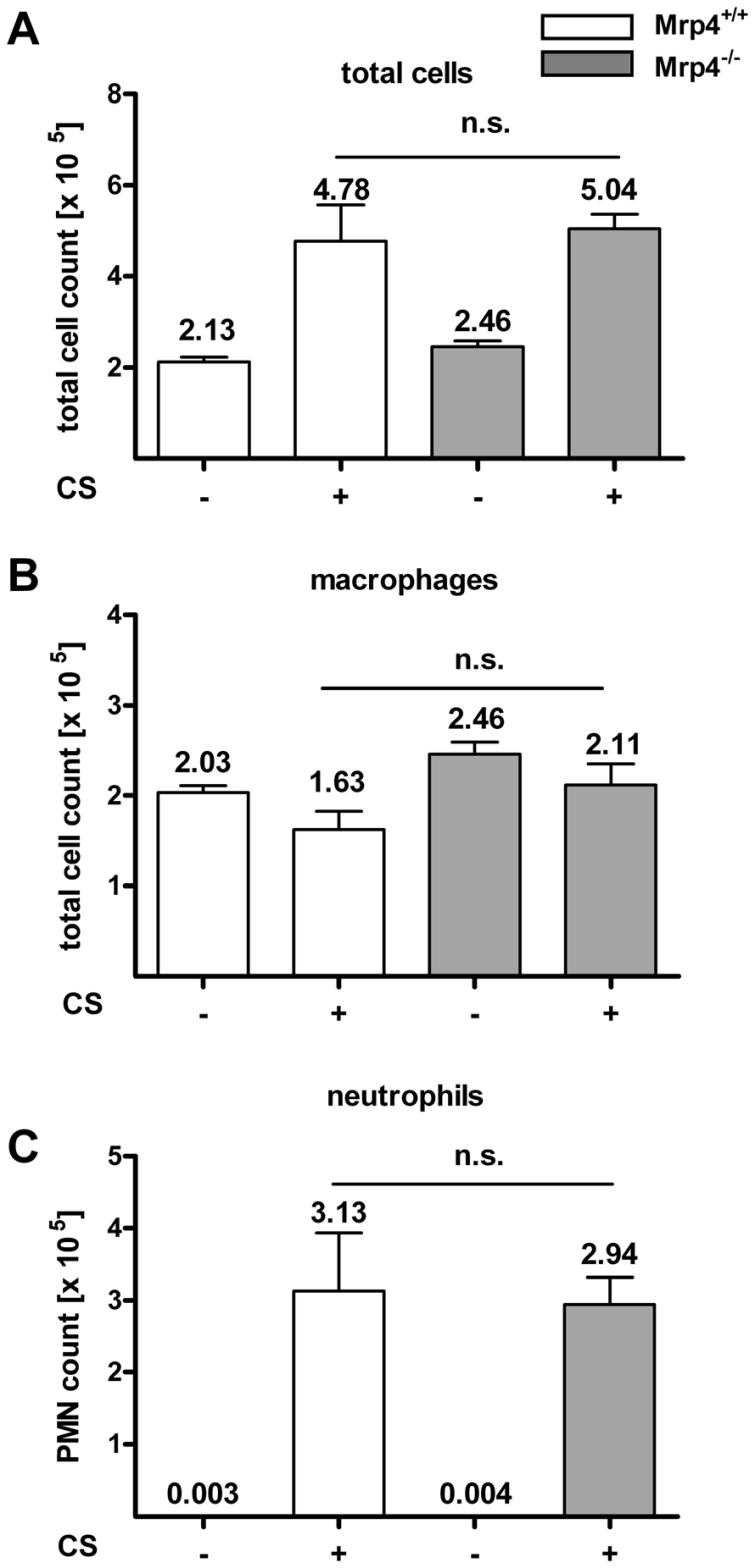
Neutrophils or macrophage concentrations were similar in BALF of *Mrp4^−/−^* and *Mrp4^+/+^* mice exposed to cigarette smoke for 4 days. A) Total cell numbers, B) macrophage numbers and C) neutrophil numbers in the BALF were measured in *Mrp4^+/+^* (white bars) and *Mrp4^−/−^* (grey bars) mice 4 days after exposure to cigarette smoke (CS, +) or fresh air as control (−). n = 8. n.s. = not significant. Data are mean ± SEM.

In a further set of *in vivo* experiments we addressed the role of Mrp4 in cigarette smoke (CS)-induced inflammatory responses. We have reported previously that in a 4-day CS model the daily application of Roflumilast led to a dose dependent reduction of neutrophils [26]. However, no positive effect on the reduction of neutrophils or macrophage recruitment was observable in *Mrp4^−/−^* mice compared to *Mrp4^+/+^* control animals (Figure 3). The application of smoke for 4 days led to an increase of the total cell number (from 2.13×10^5^ to 4.78×10^5^ cell/ml) and neutrophils (3.13×10^5^ cell/ml). Since the recruitment of macrophages into the BALF was not induced upon treatment with smoke for 4 days; the increase of total cell numbers reflects almost exclusively the recruitment of neutrophils into the lung. However, the increase of neutrophils in *Mrp4^−/−^* mice was similar to *Mrp4^+/+^* control animals suggesting that Mrp4 has no effect on CS-induced neutrophil recruitment into the lung.

### The absence of Mrp4 and the inhibition of BRCP by KO143 did not affect the release of inflammatory cytokines from LPS-stimulated whole blood

**Figure 4:**
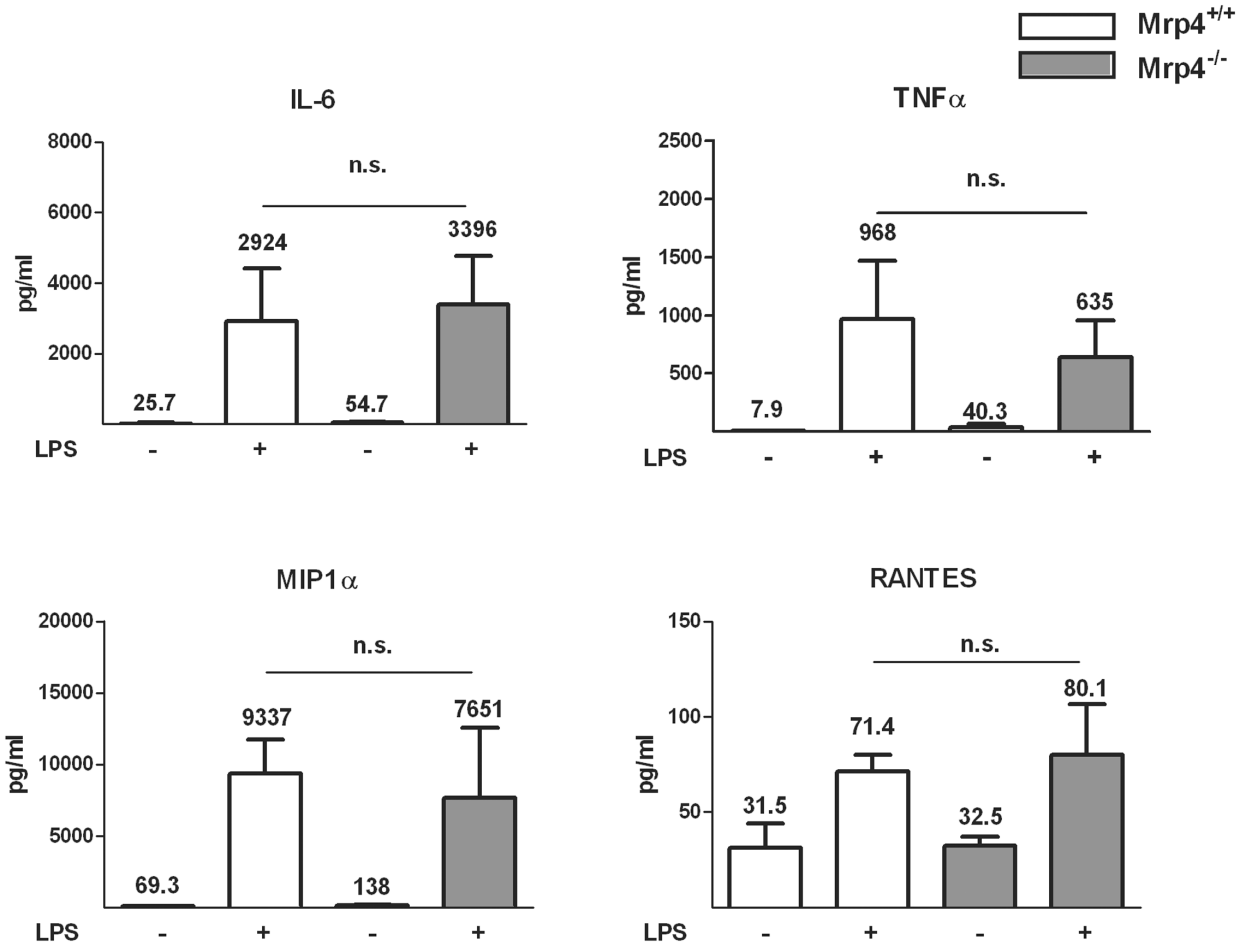
LPS-induced Cytokine release in whole blood was not dependent on Mrp4. Blood samples were collected retro-bulbar from *Mrp4^−/−^* mice (open bars) and *Mrp4^+/+^* mice (grey bars) and treated with LPS (+; 600 ng/ml) or PBS for control (−). After 4 h incubation *ex vivo* the concentrations of IL-6, TNFα, MIPα, and RANTES were measured in the plasma. n = 4–6. * p = 0.05. n.s. not significant. Data are mean ± SEM.

We tested whether the absence of Mrp4 has an impact on cytokine release into whole blood after LPS stimulation. As expected, 4 h after treatment with LPS we measured a significant increase of the cytokine concentrations of IL-6, TNFα, MIP1α and RANTES in the whole blood of *Mrp4^+/+^* mice (Figure 4). However, there was no difference detectable in the LPS-induced cytokine release between whole blood of *Mrp4^+/+^* and *Mrp4^−/−^* mice indicating that the absence of Mrp4 does not alter the inflammatory response to LPS.

**Figure 5:**
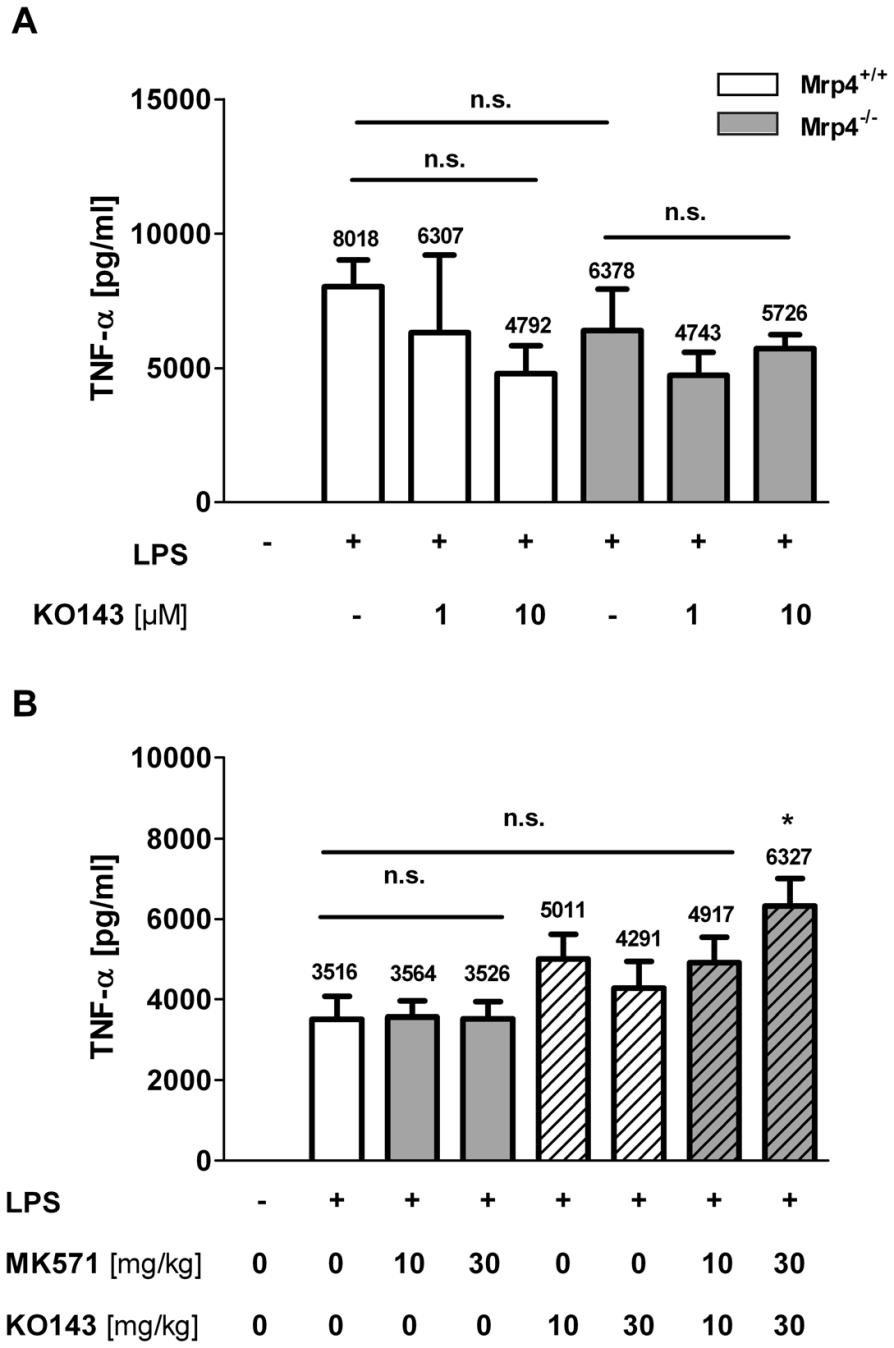
The BCRP inhibitor KO143 did not affect LPS-induced TNFα release in whole blood *in vitro* and in BALF *in vivo*. **A**) Upon incubation with different concentrations of the BCRP inhibitor KO143 (1 µM, 10 µM) or PBS for control (−) for 30 min, TNFα release in whole blood samples from *Mrp4^+/+^* mice (open bars) or *Mrp4^−/−^* mice (grey bars) was stimulated with LPS (+, 600 ng/ml) or PBS for control (−). 4 h after stimulation, blood plasma was collected and TNFα concentrations measured. Result is representative for 3 independent experiments. n = 3–4. n.s. = not significant. Data are mean ± SEM. B) TNFα release in the BALF was measured in wt mice 4 h after application of LPS (+) or PBS for control (−). Mice were pretreated with MK571 (grey bars; 10 and 30 mg/kg), KO143 (striped open bars; 10 and 30 mg/kg), a combination of MK571 and KO143 (striped grey bars; 10 and 30 mg/kg of each inhibitor) or vehicle for control (open bar). n = 8. n.s. = not significant. * p = 0.006. Data are mean ± SEM.

Our *in vivo* results clearly indicate that the recruitment of neutrophils and eosinophils is not reduced in mice lacking Mrp4. However, it is possible that other ABC transporters compensate for the deficiency of Mrp4. For example, it has been demonstrated that the breast cancer resistance protein (BCRP, ABCG2) contributes to the transport of cGMP from murine erythrocytes equally to Mrp4 [34]. Furthermore, it has been demonstrated in mice that BCRP and Mrp4 work in parallel to transport purine nucleoside analogues [35]. Using the BCRP specific inhibitor KO143 [21] we found that the treatment of whole blood from *Mrp4^−/−^* and *Mrp4^+/+^* mice with 1 or 10 µM KO143 did not affect the TNFα release (Figure 5A) suggesting that BCRP is not able to compensate for the lack of Mrp4, at least in the used *ex vivo* model.

### LPS-induced TNFα release into the BALF was not affected in wt mice treated with MK571 and KO143

We used a pharmacological *in vivo* approach with the inhibitors MK571 and Ko143 to address the question whether the inhibition of Mrp4 together with BCRP show *in vivo* efficacy in the reduction of TNFα release into the BALF of wt mice challenged with LPS. Upon treatment with LPS the TNFα concentration in the BALF increased within 4 h from undetectable to 3516 pg/ml (Figure 5B). However, the application of MK571 (10 and 30 mg/kg,) or KO143 (10 and 30 mg/kg) did not reduce TNFα release (3564 and 3526 mg/ml, or 5011 and 4291 mg/ml. respectively). Rather we found the opposite, namely that the application of KO143 alone and the combination of KO143 with MK571 (4917 mg/ml at 10 mg/kg) led to an increase of TNFα reaching significance at the combined doses of 30 mg/kg (6327 mg/ml). Similarly, the recruitment of LPS-induced neutrophils was not reduced by the single and combined application of the inhibitors (Figure 1B; data not shown). Taken together, the inhibition of Mrp4 alone as well as the combined inhibition with BCRP are not sufficient to show efficacy in a COPD related *in vivo* model.

### Rolipram-induced cAMP elevation in the plasma was dependent on Mrp4

**Figure 6:**
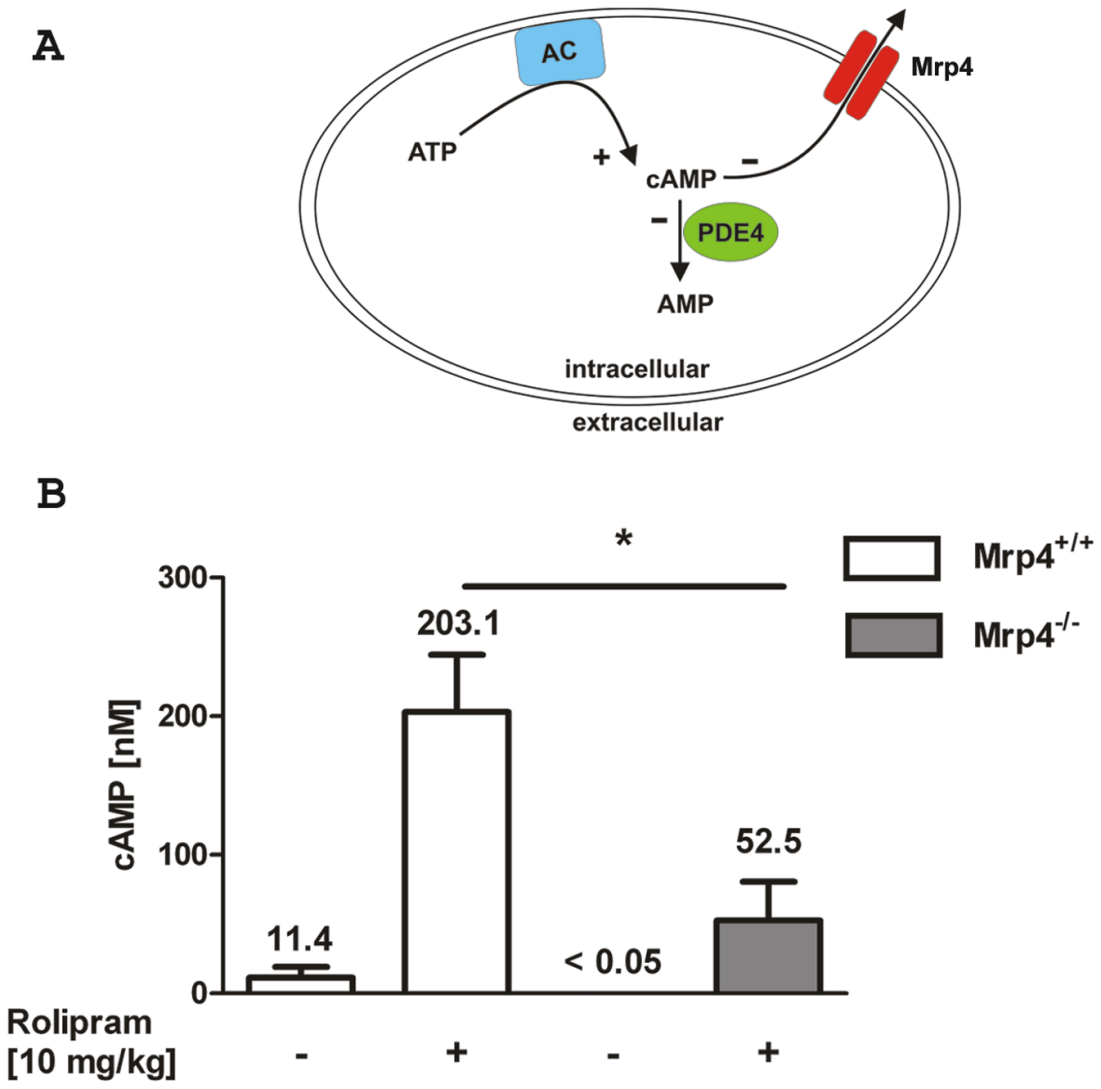
Mrp4 is involved in the control of plasma cAMP concentration after oral application of Rolipram. A) Simplified schematic of the intracellular cAMP regulation. The adenylyl cyclase (AC) catalyses the generation (+) of cAMP from ATP. cAMP is hydrolyzed (−) to inactive AMP by the activity of PDE4. Mrp4 is able to pump cAMP out of the cells (−). The inhibition of PDE4 as well as Mrp4 has the potential to increase intracellular cAMP levels. B) Rolipram (+; 10 mg/kg) or vehicle for control (−) was given orally to *Mrp4^−/−^* mice (grey bars) or *Mrp4^+/+^* mice (open bars). After 20 min blood samples were collected retro-bulbar to measure plasma cAMP concentrations. Control groups n = 3, Rolipram treated groups n = 5. * p = 0.02. Data are mean ± SEM.

The usage of the PDE4 specific inhibitor Rolipram has been reported to elevate the amount of cAMP and epinephrine, an adrenal catecholamine, into the plasma [36]. In this study the cAMP response was significantly reduced after the removal of adrenal glands indicating that the systemic increase of cAMP in this experimental setting is mainly regulated by the circulating levels of catecholamine that activate adenylyl cyclases via β2 adrenergic receptors [36]. The inhibition of PDE4 by Rolipram leads to a further increase of intracellular cAMP that is then transported rapidly out of the cell and tissues causing the rise of the extracellular cAMP level [36]. To test whether the absence of Mrp4 has an impact on the cAMP level in the plasma, we measured the cAMP concentration after oral administration of the PDE4 inhibitor Rolipram to *Mrp4^−/−^* and *Mrp4^+/+^* mice. If Mrp4 regulates cAMP levels *in vivo* (Figure 6A), a decrease in Rolipram-induced cAMP levels in the plasma of *Mrp4^−/−^* mice compared to plasma of *Mrp4^+/+^* mice would be expected.

To address this question, blood was collected from *Mrp4^−/−^* and *Mrp4^+/+^* mice 20 min after the oral administration of 10 mg/kg Rolipram. In accordance with Cheng et al. [36], the cAMP level increased in *Mrp4^+/+^* mice from 11.4±13.3 nM (in some animals below the detection level) to 203.1±92.3 nM upon treatment with Rolipram (Figure 6B). In contrast, the basal cAMP level in untreated *Mrp4^−/−^* mice was below the detection level (<0.05 nM) and the Rolipram-induced cAMP concentration was significantly lower in *Mrp4^−/−^* mice (52.5±56.4 nM). These findings indicate that Mrp4 regulates plasma cAMP concentrations *in vivo* upon Rolipram treatment. Similarly, we measured cAMP levels in the plasma 4 h after i.t. application of LPS; however, we could not detect a significant increase (data not shown). Additionally, the cAMP levels were not increased in the animal groups treated with MK571 and/or KO143 indicating that these inhibitors do not affect cAMP release upon LPS stimulation (data not shown).

## Discussion

Mrp4 has been reported to transport regulators of the immune system like eicosanoids, including the prostaglandins E_1_ and E_2_ (PGE_1_, PGE_2_), and cyclic nucleotides, including cAMP and cGMP [2]. However, the *in vivo* role of Mrp4 during acute lung inflammation is poorly defined. In this study we tested whether the absence of Mrp4 has an effect in different murine models of lung inflammation. In all models used, we could not detect any difference between *Mrp4^−/−^* and *Mrp4^+/+^* mice in respect to recruitment of neutrophils and eosinophils into the lung. Similarly, the inhibition of Mrp1/4 by MK571 did not reduce LPS-induced neutrophil recruitment. Our results are in accordance with a recent study by van de Ven et al., where the authors showed that the absence of Mrp4 did not affect adaptive immune responses after intra cutaneous injection of OVA into the ear of OVA-sensitized mice [37]. The usage of Mrp4/Mrp5-double KO mice in these sets of experiments excluded compensatory effects of Mrp5. Furthermore, a comparison of the blood counts between the whole blood from *Mrp4^−/−^/Mrp5^−/−^* mice and wild type mice did not reveal any difference indicating that Mrp4, together with Mrp5, are not involved in the differentiation of the analyzed leukocyte lineages, namely T cells, cytotoxic T cells and natural killer (NK) cells, B cells, macrophages and dendritic cells (DC) [37]. Interestingly, the same group found that Mrp4 and Mrp5 are not required for murine skin DC migration [37], whereas Mrp4 played a role for the migration of human DC toward CCL19 and CCL21 [38]. The obvious discrepancy between these two studies could be explained by a species-specific function of Mrp4 [37]. Species differences have also been described for the murine Mrp4 that showed a 100-fold higher K_m_ for the export of cGMP compared to the human protein [34].

Of note, our results do not exclude that Mrp4 may still be involved in other lung functions like the regulation of the pulmonal arterial blood pressure, the secretion of serous cell fluid and/or edema formation. Recently, it has been shown that *Mrp4^−/−^* mice are protected from the development of hypoxia-induced pulmonary arterial hypertension (PAH) [30]. The authors hypothesized that the increased intracellular cAMP in *Mrp4^−/−^* mice mediated protection against hypoxic pulmonary hypertension [30]. Furthermore, cAMP has been described to be required for serous cell fluid secretion in human airways that is regulated by the Cystic Fibrosis Transmembrane Conductance Regulator (CFTR) [39]. Interestingly, a functional association between Mrp4 and CFTR has been demonstrated in the gut epithelium [10]. However, a similar association in the lung has not been demonstrated, yet. Furthermore, the increase of cAMP by different pharmacological approaches using isoprotenerol, forskolin and prostaglandin attenuated lung edema that was associated with a critical role of cAMP in the regulation of the endothelial barrier [40]. The usage of PDE4 inhibitor Rolipram has been reported to elevate the concentration of epinephrine, an adrenal catecholamine, in the plasma of mice [36]. Subsequently, epinephrine induced an export of cAMP into the plasma that was measured 20 min upon treatment with Rolipram [36]. In this study, we showed in a set of *in vivo* experiments that Mrp4 plays a strong role in the regulation of plasma cAMP concentration under conditions of PDE4 inhibited by Rolipram. These findings suggest a potential role for Mrp4 in circulatory or vascular physiology. At this point, the biological relevance of Mrp4 in the control of plasma cAMP levels and the activation of endothelial cells, lung edema formation, CFTR regulation in the lung, serous cell fluid secretion, or PAH remain open questions. Unexpectedly, the application of the inflammatory stimulus LPS did not elevate cAMP levels in the plasma and accordingly the inhibition of Mrp4 by MK571 did not affect cAMP levels (data not shown).

We also tested whether BCRP could compensate for the absence of Mrp4, since in human erythrocytes the main transporter of cGMP is Mrp4 whereas in mice about half of the transport is mediated by BCRP [34]. However, did not find any evidence for a compensatory function of BCRP in LPS-induced TNFα release in murine whole blood. Although LPS-stimulated whole blood from Mrp4^+/+^ mice showed a slight and not significant reduction of TNFα release after the treatment with 10 µM KO143, this was not considered as a specific effect of the inhibitor, since KO143 inhibits BCRP completely at concentrations below 1 µM [22]. Nevertheless, we cannot exclude compensatory effects of other members of the Mrp family like Mrp8 that has been shown, besides Mrp4 and Mrp5, to transport both cyclic nucleotides namely cAMP and cGMP [4], [5], [41]. However, genomic expression profiling of the *Mrp4^−/−^* animals, did not reveal any up- or down-regulation of the relevant transporters and enzymes in the cAMP household (data not shown). Finally, we used the orally available inhibitors MK571 and KO143 to test whether the inhibition of BCRP is able to compensate for the inhibition of Mrp4 *in vivo*. In a five-day model the Mrp1/4 inhibitor MK571 has been demonstrated to reduce hypoxia-induced pulmonary hypertension in a dose dependent manner after daily application of 5 mg/kg or 25 mg/kg MK571 [30]. KO143 increased the oral availability of topotecan in a murine *in vivo* model at a dose of 10 mg/kg [22]. In our experiments, LPS-induced TNFα release and neutrophil recruitment was not reduced upon the application of the inhibitors at sufficient doses of 10 and 30 mg/kg. Rather the combination of MK571 with KO143 led to an increase of both parameters at a dose of 30 mg/kg. Whether this effect is related to the combined inhibition of Mrp4 and BCRP or is a compound related adverse effect is unknown. Nevertheless, we were not able to demonstrate any anti-inflammatory effect in mice after the single and combined inhibition of Mrp4 and BCRP.

It is known that the inhibition of PDE4 and the subsequent increase of intracellular cAMP is sufficient to inhibit LPS-induced release of TNFα and other inflammatory cytokines from leukocytes [42], [43]. Because, we did not detect a difference in the LPS-induced release of inflammatory cytokines from the whole blood of *Mrp4^−/−^* and *Mrp4^+/+^* mice, our studies suggest Mrp4 does not play a dominant role in the regulation of intracellular cAMP in leukocytes during acute inflammation induced by LPS. It has been described in the literature that the inhibition of PDE4 reduces the inflammatory response in different lung models [33], [44], [45]. Since Mrp4 is not expressed in neurons, inhibition of Mrp4 could reduce the effective PDE4 inhibitor concentrations and maybe the adverse effects, including the emetogenicity [46]. Likewise, in smooth muscle cells (hPASMC), where the downregulation of Mrp4 by the RNAi technique led to an additive increase of intracellular cAMP after the treatment with the PDE5 inhibitor Sildenafil [30]. Here, we tested *in vivo* whether the combination of suboptimal PDE4 inhibition in the absence of Mrp4 has an additive effect on the reduction of LPS-induced neutrophil recruitment into the lung. However, we could not detect such an additive effect.

Taken together, our *in vivo* results suggest no major role of Mrp4 in the induction of neutrophil or eosinophil recruitment into the lung of mice. Whether this is also true in humans remains to be seen since there may be differences in the biology between humans and rodents and we cannot totally rule out compensatory mechanisms in the Mrp4 deficient mice. However, our data provide no evidence for a critical role of Mrp4 in acute airway inflammation.
